# Bacteriological profile of wound swab and their antibiogram pattern in a tertiary care hospital, Saudi Arabia

**DOI:** 10.15537/smj.2022.43.12.20220681

**Published:** 2022-12

**Authors:** Azzah S. Alharbi

**Affiliations:** *From the Medical Microbiology and Parasitology Department, Faculty of Medicine, King Abdulaziz University, and from Special Infectious Agent Unit, King Fahad Medical Research Center, Jeddah, Kingdom of Saudi Arabia.*

**Keywords:** wound infection, microbial profile, Antimicrobial resistance, Saudi Arabia

## Abstract

**Objectives:**

To assess the microbial profile of wound infection and their antibiogram pattern.

**Methods:**

A retrospective study was carried out at King Abdulaziz University Hospital, Jeddah Saudi Arabia between December 2021 and July 2022 comprising data related to demographic, microbial profile and antibiotic sensitivity pattern of wound infection–suspected cases.

**Results:**

A total of 305 wound swabs were collected; of which 56.1% showed microbial growth. Among 187 microbial isolates, 62% were gram-negative bacteria, 30.5% were gram-positive bacteria and 7.5% were fungi. *Staphylococcus aureus* was the prevailing isolates 17.1%, followed by *Klebsiella pneumoniae* and *Pseudomonas aeruginosa*, each with 13.9% and Escherichia coli with 12.8 %. *Providencia sp* with 0.1% was the least isolated bacteria. Out of 173 bacterial isolates, 46.8% were sensitive to antimicrobial agents tested, while 53.2% were resistant to one and more drug tested. Of these isolates, 22% were found to be the MDR bacteria. The highest MDR percentages was noted among *Acinetobacter baumannii* (70%) followed by *Klebsiella pneumoniae* (53.9%), *Escherichia coli* (25%) and *Pseudomonas aeruginosa* (19.2%) and the least by (12.5%) by *Staphylococcus aureus.*

**Conclusion:**

The microbial isolation rates from wound infection was high, with *Staphylococcus aureus* being the most prevalent. Considerable antimicrobial resistance rate to the commonly used antibiotics was discovered. Thus, regular monitoring of microbial profile and their antimicrobial sensitivity pattern in the study region in attempt to contain antimicrobial resistance is highly recommended.

**T**he disruption in the skin’s defense barrier in term of epithelial continuity loss with or without subcutaneous tissue exposure (such as wound) creates a wet, warm, and nutrient-rich milieu that is favorable for pathogens colonization and growth.^1-3^ A wound’s progression to infection probably contributed to the numerous pathogens and host factors.^4^ A variety of microbial pathogens, including fungi, bacteria, parasites, and viruses can infect the wound.^5^ Of which, *Escherichia coli*, *Staphylococcus aureus (S. aureus)*, *Klebsiella spp. (species)*, *Pseudomonas aeruginosa*, and *Acinetobacter spp.* are among the most prevalent microorganisms isolated from both monomicrobial and polymicrobial wound infection.^4,6^ Infected wounds may hinder the healing process and lead to serious complications with substantial impact on the quality of life.^6,7^ It is among the most acquired infections at hospitals which has contributed majorly to prolonged hospitalization, and higher costs and is associated with considerable morbidity and mortality rates especially in the developing world.^6-8^ Although, the current burden of wound infection in Saudi Arabia has not been comprehensively estimated yet, it is still expected to be high. Elevated prevalence of diabetes, obesity, ischemic heart disease amongst the Saudi population as well as untrained wound care at home- which is frequently influenced by traditional medical views-may impair the healing process and raise the risk of wound infection.^9-11^ Diagnosis of the infection relies on the wound examination by an experienced clinician, which is further confirmed by infection biomarkers and microbiological analysis.^12^ While this diagnostic approach can yield useful data, it is time-consuming and dependent on the clinician’s level of expertise. Therefore, antimicrobial agents sometimes are initiated empirically which may contribute to the emergence of antimicrobial resistant (AMR) pathogens with an additional economic and clinical burden.^8^ Antimicrobial resistant pathogens poses a global health challenge particularly in developing world, where infection rate is elevated and financial constrains limit the broad usage of newer and high pricing quality assured antimicrobials.^13^ In Africa for example, therapeutic guidelines for infections depend primarily on the use of empiric antimicrobials, without support of culture results.^14^ Noting that most of the health care providers lake updated data on AMR.^15^ In Saudi Arabia, recent research has shown that antimicrobial overuse, inadequate duration of its use, and the use of broad spectrum antimicrobials are prevalent practices among physicians.^16^ Thus, wound infection caused by drug resistance pathogen is commonly reported from developing world.^8,17^ It is evident that regular monitoring of the pathogenic organisms and their antimicrobial susceptibility profile are crucial for guiding empiric antimicrobial therapy of wound infection in health institutions. Very little is currently known on the bacteria and AMR profiles of wound infection from different regions of Saudi Arabia. To fill such a gap, this study was carried out to assess the microbial profile of wound infections and their antimicrobial susceptibility pattern at King Abdulaziz University Hospital (KAUH), Jeddah. King Abdulaziz University Hospital is one of the largest governmental referral and teaching healthcare hospitals in Saudi Arabia’s Western region, with a capacity of 876 beds for diagnostic and therapeutic purposes for patients with different characteristics.^18^

## Methods

The current study utilizes a retrospective-descriptive research approach carried out between December 2021 and July 2022 in which culture results of wound swab specimens over sixth months period -from January to June 2022 -at the Microbiology Department in KAUH were retrieved. The study protocol was approved by the Research Ethics Committee at KAU, with a Reference Number of 116-22 and conducted in accordance with the Declaration of Helsinki.

Data related to demographic (age, gender, and nationality), type of microorganism involved and antibiotic sensitivity / resistance pattern of wound infection– suspected cases, were retrieved from the medical records. Patients who were taking antibiotics or had recently taken antibiotics during the previous 2 weeks at the time of sample collection were excluded. Patients presenting inadequate demography and history of antimicrobial use were excluded.

The specimens were collected from the individuals with clinical evidence of wound infections (such as; swelling, redness, pain, the presence of pus with or without odor, high grade fever and rigors) upon physician request. Prior sample collection, the edges of wound were cleaned and the surface exudates were removed by washing with physiological sterile solution, using Levine’s technique.^13^ This process is essential for the removal of environmental microbes contaminating wound surface. Samples were then aseptically obtained from wounds by rotating a sterile cotton swab under adequate pressure, without touching the nearby skin. Within 30 minutes of collection, samples were transported to a microbiological lab by reinserting swabs into test tubes filled with 0.5 mL of sterile normal saline. After that, specimens were processed and cultured following standard techniques used in medical microbiology lab.^19^ For pathogen identification, colonies formed were further processed using morphology, gram staining, and biochemical reaction.^19^ Antibiotic susceptibility testing of the detected isolates were performed using the Kirby Bauer disc diffusion method and observations were interpreted in accordance with guidelines set by the National Committee for Clinical Laboratory Standards.^20^ A pathogen that is resistant towards 2 or more classes of antibiotics is termed a multidrug-resistant pathogen (MDR).^21^ By dividing the number of susceptible/ resistant isolates by the whole number of tested isolates, the sensitivity/resistance rates of specific bacterial isolates to each tested antibiotic agent was calculated. In the case of the fungal samples, the guidelines do not require antifungal sensitivity testing as the treatment is standard and determined by the physician.

### Systematic analysis

All retrieved data were initially recorded into an Excel sheet (Microsoft Corporation, Redmond, WA) and exported to IBM SPSS Statistics for Windows, version 20 (IBM Corp., Armonk, N.Y., USA) for statistical analysis. Frequency and percentages were used to present categorical data. Chi square or fisher exact tests were performed to compare the culture positivity, proportion of bacterial isolates and resistance pattern with patients’ gender and nationality. Analysis was considered statistically significant at a *p*-value of ≤0.05.

## Results

Overall in this study, 305 wound specimens were obtained from 305 individuals who had a clinical signs of infection; of these, 45.9% were female and 54.1% were male. The study population ranged in age from 1 to 95 years, with a mean of 41.37 (SD ± 25.28) years. Approximately 31% of the samples were collected from 60 year old or more, 56.1% showed a microbial growth, while the rest were culture negative. Both female (49.7%) and males (50.2%) had nearly comparable infection rates. The incidence of microbial infections was significantly higher in the age group of 60 years old or more (67.7%), followed by the age group of

41-59 (59.4%), 19-40 (57.5%), and 0-18 (35.7%). The age, gender and nationality distribution of subjects included in this study is provided in Table 1.

**Table 1:** - Characteristics of the study population stratified by culture positivity from wound specimens (N=305).

Characteristics	Positive culture n (%)	Negative culture n (%)	Total n (%)	*P*-value
***Gender***				0.082
Female	85 (60.7)	55 (39.3)	140 (45.9)	
Male	86 (52.1)	79 (47.9)	165 (54.1)	
Total	171 (56.1)	134 (43.9)	305 (100)	
***Nationality***				0.441
Saudi	88 (55.3)	71(44.7)	159 (52.1)	
Non Saudi	83 (56.8)	63 (43.2)	146 (47.9)	
Total	171 (56.1)	134 (43.9)	305 (100)	
***Age groups (years)***				0.001*
0 to 18	25 (35.7)	45 (64.3)	70 (23.0)	
19-40	42 (57.5)	32 (42.5)	74 (24.3)	
41-59	41 (59.4)	28 (40.6)	69 (22.6)	
≥ 60	63 (67.7)	30 (32.3)	93 (30.5)	
Total	171 (56.1)	134 (43.9)	305 (100)	

Data are reported as number (%). Test used=Chi-square test. *Significant differences between groups (*p*<0.05).

### Bacterial profile

Out of 171 culture positives, 155 (90.6%) had single bacterial isolates whereas 16 (9.4%) showed a mixed growth of 2 or more of different bacteria, so total microbial isolates was 187 (Appendix 1 & 2). Among 187 microbial isolates, 116 (62%) were gram-negative bacteria, 57 (30.5%) were gram-positive bacteria and 14 (7.5%) were fungi. *Staphylococcus aureus* (*S. aureus)* was the prevailing isolates 17.1% (32/187), followed by *Klebsiella pneumoniae* and *Pseudomonas aeruginosa*, each with 13.9 % (26/187) and Escherichia coli with 12.8 % (24/187). *Providencia sp.* with 0.1% (1/187) was the least isolated bacteria Table 2. The age, gender, nationality distribution of common microbial isolates from wound are shown in Table 2

**Table 2:** - Microbial species isolated from wound infections, and their distribution stratified for gender, nationality and age of the patients.

Microbial species	Gender			Nationality		Age group			
	Total n(%)	Female n (%)	Male n (%)	Saudi n (%)	Non Saudi n (%)	0 to 18 n (%)	19-40 n (%)	41-59 n (%)	≥60 n (%)
***Gram positive bacteria***									
*Staphylococcus aureus*	32 (17.1)	18(9.6)	14(7.5)	27 (14.4)	5 (2.7)	5 (2.7)	18(9.6)	5(2.7)	4 (2.1)
*Staphylococcus epidermidis*	2 (1.1)	0 (0)	2 (1.1)	2 (1.1)	0 (0)	0 (0)	0 (0)	1(0.5)	1(0.5)
*Streptococcus agalactiae*	6 (3.2)	2 (1.1)	4 (2.1)	5 (2.7)	1(0.5)	1 (0.5)	1(0.5)	2(1.1)	2(1.1)
*Streptococcus pyogenes*	3 (1.6)	0 (0)	3 (1.6)	1 (0.5)	2 (2.7)	0 (0)	1 (0.5)	1(0.5)	1(0.5)
*Enterococcus faecalis*	8 (4.3)	3 (1.6)	5 (2.7)	4 (2.2)	4 (2.15)	1 (0.5)	1 (0.5)	3(1.6)	3(1.6)
*Enterococcus faecium*	3 (1.6)	1(0.5)	2 (1.1)	1 (0.5)	2 (1.1)	2 (1.1)	0 (0)	1 (0.5)	0 (0)
*Enterococcus gallinarum*	3 (1.6)	1(0.5)	2 (1.1)	3 (1.6)	0 (0)	0 (0)	1 (0.5)	0 (0)	2 (1.1)
*Total*	57 (30.5)	25 (13.4)	32 (17.1)	43 (23.0)	14 (7.48)	9 (4.8)	22 (11.8)	13 (7.0)	13 (7.0)
***Gram negative bacteria***									
*Klebsiella pneumoniae*	26 (13.9)	11 (5.9)	15 (8.0)	8 (4.3)	18 (9.6)	4 (2.2)	6 (3.2)	3 (1.6)	13 (7)
*Pseudomonas aeruginosa*	26 (13.9)	16 (8.6)	10 (5.4)	15 (8.0)	11(5.9)	6 (3.2)	4(2.2)	8 (4.3)	8 (4.3)
*Escherichia coli*	24 (12.8)	10 (5.4)	14 (7.5)	13 (7.0)	11(5.9)	2(1.1)	7 (3.7)	2 (1.1)	13 (7.0)
*Acinetobacter baumannii*	10 (5.3)	3 (1.6)	7 (3.5)	6 (3.2)	4 (2.1)	0(0)	1 (0.5)	5 (2.7)	4 (2.1)
*Serratia marcescens*	8 (4.3)	4 (2.2)	4 (2.2)	6 (3.2)	2 (1.1)	0 (0)	2 (1.1)	5 (2.7)	1 (0.5)
*Morganella morganii*	4 (2.1)	1(0.5)	3 (1.6)	2 (1.1)	2 (1.1)	0 (0)	0 (0)	1 (0.5)	3 (1.6)
*Enterobacter cloacae*	6 (3.2)	3 (1.6)	3(1.6)	0 (0)	6 (3.2)	2 (1.1)	1 (0.5)	1 (0.5)	2(1.1)
*Enterobacter aerogenes*	2 (1.1)	2(1.1)	0 (0)	0 (0)	2 (1.1)	0 (0)	2 (1.1)	0 (0)	0 (0)
*Citrobacter freundii*	3 (1.6)	0 (0)	3 (1.6)	0 (0)	3 (1.6)	0 (0)	0 (0)	1(0.5)	2 (1.1)
*Proteus mirabilis*	3 (1.6)	2 (1.1)	1(0.5)	2 (1.1)	1 (0.5)	0 (0)	0 (0)	1 (0.5)	2 (1.1)
*Stenotrophomonas maltophilia*	3 (1.6)	2 (1.1)	1 (0.5)	0 (0)	3 (1.6)	1 (0.5)	0 (0)	0 (0)	2 (1.1)
*Providencia sp.*	1 (0.5)	1 (0.5)	0 (0)	0 (0)	1 (0.5)	0 (0)	0 (0)	1 (0.5)	0 (0)
Total	116 (62.0)	55 (29.4)	61 (32.6)	52 (27.8)	64 (34.2)	15 (8.0)	23 (12.3)	28 (14.9)	50 (26.7)
Fungi	14 (7.5)	10 (5.4)	4 (2.2)	5 (2.7)	9 (4.8)	2 (1.1)	0 (0)	2 (1.1)	10 (5.4)
Total	187 (100)	90 (48.1)	97(51.9)	100 (53.5)	87 (46.5)	26 (13.9)	45 (24.0)	43 (23.0)	73 (39.0)

Data reported as number and percentages (%). Test used=fisher exact test, *p*-value of 0.215 with gender, <0.001 with nationality and age groups, sp.- species

### Antimicrobial profile

Species-specific resistance analysis showed that *S. aureus* isolates were relatively sensitive to oxacillin, erythromycin, ciprofloxacin and trimethoprim-sulfamethoxazole with resistance rate of 29%, 22.6%, 12.5% and 12.5. However, *S. aureus* was sensitive to clindamycin with a low resistance rate (3.2%). The majority of *Klebsiella pneumoniae* isolates were resistant to meropenem and imipenem (92.3%, each), ciprofloxacin (68%), and amikacin (66.7%). On the contrary, *Klebsiella pneumoniae* was susceptible to Piperacillin tazobactam, Amoxicillin/clavulanic acid and cefazoline with 12.5% resistance each. Of 24 tested *Pseudomonas aeruginosa* isolates, resistant to ciprofloxacin and Piperacillin tazobactam were seen with rate of 37.5% and 29%, however, only 16.7% showed a resistance to gentamicin. Most of *Escherichia coli* were resistant to ciprofloxacin (81%). A 100% resistance towards meropenem, imipenem, cefepime and ceftazidime were seen in *Acinetobacter baumannii*. All isolates of *Streptococcus agalactiae* (n=6) and S. pyogenes (n=3) were sensitive to all tested antibiotics (clindamycin, erythromycin and penicillin). All isolates of Enterobacter aerogenes (n=2) and Citrobacter freundii (n=3) were sensitive to Ciprofloxacin, Gentamicin and trimethoprim-sulfamethoxazole while the isolates of Stenotrophomonas maltophilia (n=3) were all sensitive to trimethoprim-sulfamethoxazole. A detailed overview of antibiotic sensitivity pattern of both gram positive and gram negative bacteria are presented in Table 3 and Table 4.

**Table 3:** - Antimicrobial profile of gram-positive bacterial isolates.

Antibiotics	*S. aureus* n=(32)			*S. epidermidis* n=(32)			*E. faecalis* n=(32)			*E.f aecium* n=(32)			*E. gallinarum* n=(32)		
	T	R	S	T	R	S	T	R	S	T	R	S	T	R	S
Ciprofloxacin	32	4 (12.5)	28 (87.5)	2	1 (50.0)	1 (50.0)	-	-	-	-	-	-	-	-	-
Trimethoprim-Sulfamethoxazole	32	4 (12.5)	28 (87.5)	2	0 (0)	2 (100)	-	-	-	-	-	-	-	-	-
Clindamycin	31	1 (3.2)	30 (96.8)	2	0 (0)	2 (100)	-	-	-	-	-	-	-	-	-
Erythromycin	31	7 (22.6)	24 (77.4)	2	1 (50.0)	1 (50.0)	-	-	-	-	-	-	-	-	-
Oxacillin	31	9 (29.0)	22 (71.0)	2	2 (100)	0 (0)	-	-	-	-	-	-	-	-	-
Gentamycin	-	-	-	-	-	-	6	2 (33.3)	4 (66.7)	2	1 (50.0)	1 (50.0)	3	0 (0)	3 (100)
Vancomycin	12	0 (0)	12 (100)	1	0 (0)	1 (50)	-	-	-	3	2 (66.7)	1 (33.3)	3	2 (66.7)	1 (33.3)
Ampicillin	-	-	-	-	-	-	7	0 (0)	7 (100)	3	3 (100)	0 (0)	3	0 (0)	3 (100)
Linezolid	-	-	-	-	-	-	-	-	-	2	1 (50)	1 (50.0)	2	0 (0)	2 (100)
Piperacillin tazobactam	-	-	-	-	-	-	2	0 (0)	2 (100)	-	-	-	-	-	-

Data are reported as number and percentage (%). T: total number of isolates tested against each antibiotic, R: number of isolates resistance to antibiotic, S: number of isolates sensitive to antibiotic, - : not done

**Table 4:** - Antimicrobial profile of Gram-negative bacterial isolates.

Antibiotics	*Klebsiella pneumonia* n=(26)			*Pseudomonas aeruginosa* n=(26)			*Escherichia coli* n=(24)			*Acinetobacter baumannii* n=(10)			*Serratia marcescens* n=(8)		
	T	R	S	T	R	S	T	R	S	T	R	S	T	R	S
Meropenem	13	12 (92.3)	1 (7.7)	9	6 (66.7)	3 (33.3)	4	0 (0)	4 (100)	7	7 (100)	0 (0)	-	-	-
Amikacin	9	6 (66.7)	3 (33.3)	4	4 (100)	0 (0)	9	0 (0)	9 (100)	5	2 (40.0)	3 (60.0)	-	-	-
Ciprofloxacin	25	17 (68.0)	8 (32)	24	9 (37.5)	15 (62.5)	21	17 (81)	4 (19)	10	7 (70.0)	3 (30.0)	8	2 (25.0)	6 (75.0)
Gentamicin	24	10 (41.7)	14 (58.3)	24	4 (16.7)	20 (83.3)	24	8 (33.3)	16 (66.7)	7	1 (14.3)	6 (85.7)	8	0 (0)	8 (100)
Imipenem	13	12 (92.3)	1 (7.7)	8	6 (75)	2 (25)	4	0 (0)	4 (100)	7	7 (100)	0 (0)	-	-	-
Trimethoprim-sulfamethoxazole	25	15 (60.0)	10 (40.0)	-	-	-	24	10 (41.7)	14 (58.3)	10	7 (70)	3 (30)	8	2 (25)	6 (75.0)
Ertapenem	11	6 (54.5)	5 (45.5)	-	-	-	12	0 (0)	12 (100)	-	-	-	4	0 (0)	4 (100)
Cefepime	1	1 (100)	0 (0)	5	5 (100)	0 (0)	2	0 (0)	2 (100)	7	7 (100)	0 (0)	8	0 (0)	8 (100)
Piperacillin tazobactam	8	1 (12.5)	7 (87.5)	24	7 (29.0)	17 (71.0)	11	1 (9.0)	10 (91.0)	10	7 (70.0)	3 (30.0)	-	-	-
Amoxicillin/clavulanic acid	8	1 (12.5)	7 (87.5)	-	-	-	11	4 (36.4)	7 (63.6)	-	-	-	-	-	-
Cefazoline	8	1 (12.5)	7 (87.5)	-	-	-	9	3 (33.3)	6 (66.7)	3	0 (0)	3 (100)	-	-	-
Tigecycline	3	3 (100)	0 (0)	-	-	-	-	-	-	1	0 (0)	1 (100)	-	-	-
Ceftazidime	-	-	-	24	5 (20.8)	19 (79.2)	1	1(100)	0 (0)	7	7 (100)	0 (0)	-	-	-
Cefuroxime	1	-	1 (100)	-	-	-	4	3 (75)	1 (25)	-	-	-	-	-	-

Data reported as number and percentage (%). T: total number of isolates tested against each antibiotic, R: number of isolates resistance to antibiotic, S: number of isolates sensitive to antibiotic, – represents ‘not done’

**Table 4:** - Antimicrobial profile of Gram-negative bacterial isolates (continuation).

Antibiotics	*Morganella morganii* n=(4)			*Enterobacter cloacae* n=(6)			*Proteus mirabilis* n=(3)			*Providencia sp.* n=(1)		
	T	R	S	T	R	S	T	R	S	T	R	S
Meropenem	-	-	-	1	1 (100)	0 (0)	-	-	-	-	-	-
Amikacin	1	0 (0)	1 (100)	1	1 (100)	0 (0)	-	-	-	1	0 (0)	1 (100)
Ciprofloxacin	4	3 (75.0)	1 (25.0)	6	2 (33.3)	4 (66.7)	2	1 (50.0)	1 (50.0)	1	1 (100)	0 (0)
Gentamicin	4	0 (0)	4 (100)	6	1 (16.7)	5 (83.3)	2	0 (0)	2 (100)	1	1 (100)	0 (0)
Imipenem	-	-	-	1	1 (100)	0 (0)	-	-	-	-	-	-
Trimethoprim-sulfamethoxazole	4	2 (50.0)	2 (50.0)	6	2 (33.3)	4 (66.7)	2	1 (50.0)	1 (50.0)	1	1 (100)	0 (0)
Ertapenem	1	0 (0)	1 (100)	3	1 (33.3)	2 (66.7)	-	-	-	1	0 (0)	1 (100)
Cefepime	4	0 (0)	4 (100)	5	0 (0)	5 (100)	-	-	-	1	0(0)	1 (100)
Piperacillin tazobactam	-	-	-	-	-	-	1	0 (0)	1 (100)	-	-	-
Amoxicillin/clavulanic acid	-	-	-	-	-	-	1	0 (0)	1 (100)	-	-	-
Cefazoline	-	-	-	-	-	-	1	0 (0)	1 (100)	-	-	-
Tigecycline	-	-	-	-	-	-	-	-	-	-	-	-
Ceftazidime	-	-	-	-	-	-	-	-	-	-	-	-
Cefuroxime	-	-	-	-	-	-	-	-	-	-	-	-

Data reported as number and percentage (%). T: total number of isolates tested against each antibiotic, R: number of isolates resistance to antibiotic, S: number of isolates sensitive to antibiotic, – represents ‘not done’, sp.: species

### Multi drug resistant pattern

Out of 173 bacterial isolates, 81 (46.8%) were sensitive to antimicrobial agents tested, while 92 (53.2%) were resistant to one and more drug tested. Of these isolates, 38 (22%) were found to be the MDR bacteria. The overall MDR rate among gram-negative bacterial isolates 29.3% (34/116) was higher than gram positive ones 7% (4/57). The highest MDR percentages was noted among *Acinetobacter baumannii (A. baumanii)* (70%) followed by *Klebsiella pneumoniae* (53.9%), *Escherichia coli* (25%), and *Pseudomonas aeruginosa* (19.2%) and the least by (12.5%) by *S. aureus* Table 5. Microbial resistance was not statistically significantly different by patient age (*p*=0.192), gender (*p*=0.625) and nationality (*p*=0.101) Table 6.

**Table 5:** - Distribution of bacterial isolates stratified by number of resistances.

Microbial species	Number of resistance								
	Total	R_0_	R_1_	R_2_	R_3_	R_4_	R_5_	R_≥6_	MDR
*Gram positive bacteria*									
*Staphylococcus aureus*	32 (17.1)	18 (56.2)	7 (21.9)	3 (9.4)	4 (12.5)	-	-	-	4 (12.5)
*Staphylococcus epidermidis*	2 (1.1)	-	-	2(100)	-	-	-	-	-
*Streptococcus agalactiae*	6 (3.2)	6 (100)	-	-	-	-	-	-	-
*Streptococcus pyogenes*	3 (1.6)	3 (100)	-	-	-	-	-	-	-
*Enterococcus faecalis*	8 (4.3)	6 (75.0)	2 (25.0)	-	-	-	-	-	-
*Enterococcus faecium*	3 (1.6)	-	1(33.3)	1(33.3)	-	1 (33.3)	-	-	-
*Enterococcus gallinarum*	3 (1.6)	1 (33.3)	2 (66.7)	-	-	-	-	-	-
*Total*	57 (30.5)	34 (59.7)	12 (21.1)	6 (10.5)	4 (7.0)	1 (1.75)	-	-	4 (7.0)
***Gram negative bacteria***									
***Klebsiella pneumoniae***	26 (13.9)	7 (26.9)	2 (7.7)	3 (11.5)	2 (7.7)	5 (19.2)	3 (11.5)	4 (15.4)	14 (53.9)
***Pseudomonas aeruginosa***	26 (13.9)	14 (53.8)	4 (15.4)	3 (11.5)	1 (3.8)	-	-	4 (15.4)	5 (19.3)
***Escherichia coli***	24 (12.8)	5 (20.8)	6 (25.0)	7 (29.2)	3 (12.5)	-	-	3 (12.5)	6 (25.0)
*Acinetobacter baumannii*	10 (5.3)	3 (30.0)	-	-	-	-	-	7 (70.0)	7 (70.0)
***Serratia marcescens***	8 (4.3)	4(50.0)	4 (50.0)	-	-	-	-	-	-
*Morganella morganii*	4 (2.1)	1(25.0)	1(25.0)	2 (50.0)	-	-	-	-	-
***Enterobacter cloacae***	6 (3.2)	3 (50.0)	-	2 (33.3)	-	1 (16.7)	-	-	1(16.7)
***Enterobacter aerogenes***	2 (1.1)	2 (100)	-	-	-	-	-	-	-
*Citrobacter freundii*	3 (1.6)	3 (100)	-	-	-	-	-	-	-
***Proteus mirabilis***	3 (1.6)	2 (66.7)	-	1 (33.3)	-	-	-	-	-
***Stenotrophomonas maltophilia***	3 (1.6)	3 (100)	-	-	-	-	-	-	-
*Providencia sp.*	1 (0.1)	-	-	-	1 (100)	-	-	-	1 (100)
Total	116 (62.0)	47 (40.6)	17 (14.7)	18 (15.5)	7 (6)	6 (5.2)	3 (2.6)	18 (15.5)	34 (29.3)

Values are presented as number and percentage (%). The total number of the microbial isolates is more than the number of samples duo to the polymicrobial infection. R0: no resistance against antimicrobial agents, R1: resistance for 1 class of antimicrobial agents, R2: resistance for 2 classes of antimicrobial agents, R3: resistance for 3 classes of antimicrobial agents, R4: resistance for 4 classes of antimicrobial agents, R5: resistance for 5 classes of antimicrobial agents, R≥6: resistance for 6 or more classes of antimicrobial agents, MDR: multidrug-resistant pathogen

**Table 6:** - Distribution of microbial resistance stratified for gender, nationality and age of the patients.

Characteristics		Number of resistance								
	Total	R_0_	R_1_	R_2_	R_3_	R_4_	R_5_	R_≥6_	MDR	*P*-value
***Gender***										0.192
Female	90 (48.1)	51 (53.7)	9 (31)	9 (37.5)	8 (72.7)	3 (42.9)	1 (33.3)	9 (50.0)	21 (23.3)	
Male	97 (51.9)	44 (46.3)	20 (69.0)	15 (62.5)	3 (27.3)	4 (57.1)	2 (66.7)	9 (50.0)	18 (18.6)	
Total	187 (100)	95 (100)	29 (100)	24 (100)	11 (100)	7(100)	3 (100)	18 (100)	39 (20.9)	
***Nationality***										0.188
Saudi	100 (53.5)	53 (55.8)	20 (69)	11 (45.8)	5 (45.5)	1(14.3)	1 (33.3)	9 (50.0)	16 (16.0)	
Non Saudi	87 (46.5)	42 (44.2)	9 (31)	13 (54.2)	6 (54.5)	6 (85.7)	2 (66.7)	9 (50.0)	23 (26.4)	
Total	187 (100)	95 (100)	29 (100)	24 (100)	11 (100)	7 (100)	3 (100)	18 (100)	39 (20.9)	
***Age groups (years)***										0.101
0 to 18	26 (13.9)	19 (20)	3 (10.3)	1 (4.2)	0 (0)	3 (42.9)	0 (0)	0 (0)	3 (11.5)	
19-40	46 (24.6)	22 (23.2)	7 (24.1)	6 (25)	4 (36.4)	1 (14.3)	1 (33.3)	5 (27.8)	11 (24.0)	
41-59	44 (23.5)	20 (21.1)	12 (41.4)	5 (20.8)	1 (9.1)	2 (28.6)	0 (0)	4 (22.2)	7 (16.0)	
≥60	71 (38.0)	34 (35.8)	7 (24.1)	12 (50.0)	6 (54.5)	1 (14.3)	2 (66.7)	9 (50.0)	18 (25.3)	
Total	187 (100)	95 (100)	29 (100)	24 (100)	11 (100)	7 (100)	3 (100)	18 (100)	39 (20.9)	

Data reported as number (%); Test used= Chi-square test.,R0, no resistance against antimicrobial agents; R1, resistance for 1 class of antimicrobial agents; R2, resistance for 2 classes of antimicrobial agents; R3, resistance for 3 classes of antimicrobial agents; R4, resistance for 4 classes of antimicrobial agents; R5, resistance for 5 classes of antimicrobial agents;R≥6, resistance for 6 or more classes of antimicrobial agents; The total number of the microbial isolates is more than the number of samples duo to the polymicrobial infection.

## Discussion

Infections of the wound can prolong hospitalization and increase mortality rates by 70–80%.^22^ Clinical management of such infections are based on 2 essential factors, antibiotic therapy and wound care.^23^ The antibiotic administration is usually initiated empirically, which possibly contributes to the development of antimicrobial resistant pathogens.^8^ In developing countries, Saudi Arabia in particular, periodic analysis of the local epidemiology and antimicrobial susceptibility pattern of the involved pathogens-often underestimated- is required for effective application of empirical therapy and limiting the spread of antibiotic resistance. As part of routine microbiology laboratory analysis, culture methods are primarily used to identify and isolate potential microorganisms from swabs, and other types of specimens to determine their species and antimicrobial sensitivities, as a guide for effective therapy.

In the current retrospective analysis, 20 microbial species were recovered from 171 patients with clinical evidence of wound infection, yielding a 56.1% isolation rate. These results match those observed in previous studies from Nepal (57.4%), Bahir Dar (53%), and Gondar (52%).^13,24^ It seems apparent that infection of the wound poses a significant clinical concern. In most of cases (90.6%), only single bacterial species dominated the wounds’ microbial population. These results reflect those of Mohammed et al,^24^ Upereti et al,^25^ KC et al^26^ and Maharjan et al^5^ who also found that single bacterial species colonized 81.7%, 97.3%, 98%, and 96.1% of wounds culture. The proportion of polymicrobial infection observed in this investigation is far below those observed by Yeong et al,^27^ who reported a higher wound prevalence of polymicrobial resistant bacteria, but they are broadly consistent with earlier research.^5,24,26^

Infection levels were highest among patients over the age of 60 (69.6%), followed by those aged 41-59 (59.5%). This may be due to age related alterations in both arm of immunity, the innate and adaptive immune systems, which reduce their ability to combat infection.^28^ Gram-negative bacteria were more prevalent (62%) than Gram-positive bacteria (30.5%), supporting the findings of earlier research in Saudi Arabia and other countries.^8,29-32^ However, *Staphylococcus aureus* was the predominant isolate followed by *Pseudomonas aeruginosa*, *Klebsiella pneumoniae,* and *Escherichia coli*. This trend is in agreement with those reported by El-Saed et al,^29^ Shimekaw et al,^13^ Rai et al^22^ and others.^4,5,33^ This result may be explained by the fact that most of these microbial isolates are part of skin and gut normal flora, so they are easily spread when there are breaks or cuts in the skin or soft tissue. Another possible explanation for this is that these isolates frequently found in health care environment as a contaminant.^4,34^

Presently, 22% of bacterial isolates were multi drug resistant. This is in agreement with the earlier study in which MDR bacteria account for 14%-22% of wound infection, however much lower than that of previously reported rates from Ethiopia with 76.1% -95.5%, and Bangladesh 66-69%.^17,24,33,34^ This is may be due to variations in type of isolated pathogens, characteristics of study population, insufficient access to effective medications, ineffective treatment plans, low treatment adherence, poorly managed infection control programs, as well as irrational and inappropriate use of antimicrobial medications in these countries.^35^ This is maybe due to variations in type of isolated pathogens, characteristics of the study population as well as irrational and inappropriate use of antimicrobial medications in these countries of multiple sectors at Saudi Arabia to limit the growth, spread, and emergence of MDR pathogens.^36^

Regarding species specific MDR pattern, 70% of *A. baumanii* and 53.9% of *Klebsiella pneumoniae* showed MDR followed by *Escherichia coli* (25%), *Pseudomonas aeruginosa* (19.2%) and *Staphylococcus aureus* (12.2%). This trend of species specific MDR profile was lower when compared to those reported from other developing countries where the close monitoring and tracking of antimicrobial resistance is questionable.^17,24,33,37^ This study found that prevalence of MDR microbial isolates was independent of age, gender, and nationality of patients. The results obtained showed a high resistance rates of the common isolated gram negative bacteria to meropenem, imipenem, cefepime and ceftazidime with (93-100%), ciprofloxacin (68-81%), and amikacin (66.7%). In particular, *Klebsiella pneumoniae* demonstrated a highest resistance towards meropenem, imipenem (92.3%, each), ciprofloxacin (68%), and amikacin (66.7%). This outcome is contrary to that of Tarana et al^4^ who reported a high sensitivity of klebsiella to imipenem (83.3%) and amikacin (66.7%). Sisay et al^38^ (2019) stated in their systematic review that *Escherichia coli* exhibited a relatively low resistance rate towards ciprofloxacin (27%). This differs from the findings presented here where 81% of *Escherichia coli* were resistant to ciprofloxacin. A 100% *A. baumanii* showed a resistance towards meropenem, imipenem, cefepime and ceftazidime. These finding are partially confirmed by other studies which showed the resistance of *A. baumanii* towards amikacin in 70.6% and imipenem in 83.3%,^8,39^ however, the study of Puca et al^8^ and Guan et al^30^ was highly sensitive to amikacin (96.7%) and imipenem (100%). The observed disparity in bacterial susceptibility profile could be related to the variation in the level of irrational antibiotic use. Thus, precise comparisons of antibiotic susceptibility profile across different nations are difficult.

### Study limitations

Since the study was a retrospective, an in-depth data on the patients profile was not available due to the improper documentation and storage. As the data about the patient’s pathologies were missing. Numbers tested for some bacterial isolates and antibiotic combinations were small, limiting interpretation. Moreover, the study was single centred carried out in a small size of sample and for a short period of time, which was another limitation. However, a comprehensive work-up of pathogenic isolates and antimicrobial sensitivity profiles for wound infections in our institution were developed, which can be used as a guide for the appropriate usage of empiric antimicrobial therapy. Including more samples in a multicenter study would have yielded more significant results.

In conclusion, the microbial isolation rate from wound infection was high. The prevailing microbial isolates in the present study were *Staphylococcus aureus*, *Klebsiella pneumoniae,* *Pseudomonas aeruginosa* and *Escherichia coli*. Gram-negative wound pathogens are isolated at higher rates than Gram-positive ones. High resistance to one or more of antimicrobial agents was reported with a considerable proportion of them displayed MDR. Therefore, periodic surveillance of microbial profile and their antimicrobial sensitivity pattern in the study region is essential for efficient wound infection management with appropriate antibiotics, in attempt to contain antimicrobial resistance.

## Appendix

**Appendix 1:** - Microbial species isolated from wound infections.

*Microbial species*	n (%)
***Gram positive bacteria***	
*Staphylococcus aureus*	32 (17.1)
*Staphylococcus epidermidis*	2 (1.1)
*Streptococcus agalactiae*	6 (3.2)
*Streptococcus pyogenes*	3 (1.6)
*Enterococcus faecalis*	8 (4.3)
*Enterococcus faecium*	3 (1.6)
*Enterococcus gallinarum*	3 (1.6)
*Total*	57 (30.5)
***Gram negative bacteria***	
*Klebsiella pneumoniae*	26 (13.9)
*Pseudomonas aeruginosa*	26 (13.9)
*Escherichia coli*	24 (12.8)
*Acinetobacter baumannii*	10 (5.3)
*Serratia marcescens*	8 (4.3)
*Morganella morganii*	4 (2.1)
*Enterobacter cloacae*	6 (3.2)
*Enterobacter aerogenes*	2 (1.1)
*Citrobacter freundii*	3 (1.6)
*Proteus mirabilis*	3 (1.6)
*Stenotrophomonas maltophilia*	(1.6)
*Providencia specie*	1 (0.1)
*Total*	116 (62.0)
***Polymicrobial 1***	
*Mixed gram-positive/gram-negative bacteria*	16 /171 (9.4)
*Fungi*	14 (7.5)
*Total*	187 (100)

The total number of the microbial isolates is more than the number of samples duo to the polymicrobial infection, where Mixed gram-positive/gram-negative bacteria have been detected.1 Details of polymicrobial infections are shown in Appendix 2.

**Appendix 2:** - Details of polymicrobial infections.

*Polymicrobial*	n (%)
*Escherichia coli* and Gram-negative bacteria	2 (12.5)
*Escherichia coli* and Gram-positive bacteria	4 (25.0)
*Pseudomonas aeruginosa* and Gram-positive bacteria	4 (25.0)
*Pseudomonas aeruginosa* and Gram-negative bacteria	2 (12.5)
Mixed Gram-positive and negative bacteria	3 (18.8)
Mixed Gram-positive bacteria	1 (6.3)
Total	16 (100)

Values are presented as number and percentages (%).
